# Unfolded Protein Response in Lung Health and Disease

**DOI:** 10.3389/fmed.2020.00344

**Published:** 2020-07-30

**Authors:** Nektarios Barabutis

**Affiliations:** School of Basic Pharmaceutical and Toxicological Sciences, College of Pharmacy, University of Louisiana Monroe, Monroe, LA, United States

**Keywords:** endothelial dysfunction, ER stress, P53, inflammation, vasculature

## Abstract

The unfolded protein response (UPR) is a complex element, destined to protect the cells against a diverse variety of extracellular and intracellular challenges. UPR activation devises highly efficient responses to counteract cellular threats. If those activities fail, it will dictate cellular execution. The current work focuses on the role of UPR in pulmonary function, by immersing into the highly interrelated network that operates toward the endothelial barrier function. A highly sophisticated UPR manipulation shall reveal new therapeutic possibilities against inflammatory lung disease, such as acute lung injury and acute respiratory distress syndrome.

## Unfolded Protein Response

The ribosomes of the endoplasmic reticulum (ER) produce secretory proteins, which are consequently subjected to modifications by cellular chaperones. The excessive abundance of misfolded proteins in the intracellular niche induces ER stress (1), which in turn induces the activation of the unfolded protein response (UPR) (2). This molecular machinery is in charge of protein synthesis, trafficking, and folding able to promote both cell survival and death (3). In the case of mild ER stress, UPR initiates survival responses (4). Upon severe circumstances, it will execute the cells by triggering lethal mechanisms (5–7).

Protein kinase RNA-like ER kinase (PERK), activating transcription factor 6 (ATF6), and the inositol-requiring enzyme-1α (IRE1α) are the major components of the UPR (8, 9). The binding immunoglobulin protein (BiP) is a member of the heat shock protein (HSP) 70, and it is destined to assist in the maturation and folding of nascent proteins (10). When those proteins bind to the BiP, they are inactive. Indeed, the release of this protein will capacitate UPR (11).

Our genome encodes for IRE1α and IRE1β. IRE1α is expressed ubiquitously, and it is essential for mice. IRE1α knockout mice exhibit embryonic lethality. On the contrary, IRE1β knockout mice are viable (12–14). IRE1 associates with BiP when there is no evident ER stress. Upon situations of such stress, BiP will relocate from IRE1 to the misfolded ER protein and will initiate auto-phosphorylation and dimerization (15). Consequently, IRE1α will cleave the X-box binding protein-1 (XBP1). This modification produces XBP1S, which is considered the active XBP1 form, strongly involved in ER membrane biogenesis, folding, and secretion (16).

PERK is structurally related to IRE1α and exists as a monomer under unstressed conditions (17). In the ER, the excessive abundance of proteins which are not folded correctly activates PERK via dimerization and auto-phosphorylation (18). This modification will phosphorylate (I) the eukaryotic translation initiation factor 2-alpha (elf2a) and (II) the nuclear factor erythroid 2-related factor 2 (nrf2). All those events will activate ATF4 and nrf2 (19). PERK may also be activated by tau accumulation (20).

ATF6 is a type 2 transmembrane transcription factor. ER stress initiates the release of BiP from ATF6 (9), which in turn will be cleaved by serine protease site-1, as well as by metalloprotease site-2. Those modifications will result in its activation (21). It has been reported that ATF6 isoforms are essential for life in certain organisms. Silencing of ATF6α and ATF6β has been shown to be associated with lethal outcomes in nonhuman vertebrates. Moreover, genetic manipulations of the ATF6 cause crucial problems in the human eye, associated with dysfunctions in neuroretina and blindness (22).

UPR is considered a promising target for cardiovascular disease therapy. There are two different strategies to employ UPR toward the development of new drugs. The first method suggests to activate the adaptive pathway of UPR, so as to counteract ER stress. The second one indicates to suppress the proapoptotic components of UPR to promote cell survival (23). The current knowledge regarding the exact interrelations between the UPR components is incomplete, and it is speculated that such investigations may help utilize the UPR-mediated survival mechanisms to oppose cardiovascular disease (16, 24). In the following sections of our manuscript, we will refer to the role of UPR in both lung disease and health, in order to seed light to those events.

## UPR Induction Promotes Lung Disease

In pulmonary fibrosis, IRE1a kinase/RNase suppression exerts anti-fibrotic effects in the lungs. The exposure of murine lungs to bleomycin caused ER stress, as reflected in the activation of IRE1a and the consequent development of pulmonary fibrosis. The small-molecule IRE1α kinase-inhibiting RNase attenuator (KIRA) 7 and 8 prevented lung fibrosis; thus, those observations revealed that IRE1α is probably a therapeutic target for lung fibrosis (25).

The activation of PERK has been associated with lung fibroblast differentiation due to endothelin 1 (ET-1) or thrombin. In WI-38 human cells, which are embryonic lung fibroblasts, ET-1 and thrombin induced the expression of the UPR markers PERK and BiP. Moreover, they augmented the levels of α-smooth muscle actin (α-SMA) and collagen (I, IV) and activated the c-Jun N-terminal kinase (JNK). Those effects were diminished by PERK knockdown. Similar observations were observed in an *in vivo* study of bleomycin-induced pulmonary fibrosis (26).

Inhibition of the IRE1α signaling pathway blocked ER expansion via the XBP-1-dependent pathway, as well as the activation of myofibroblasts by the tumor growth factor β. Moreover, it lessened the fibrosis in both the liver and the skin, reverted the fibrotic effects in myofibroblasts, and affected the suppressive effects of miR-150 in α-SMA expression (27).

The lungs of patients with idiopathic pulmonary fibrosis are subjected to increased ER stress, and investigations aimed to examine whether ER stress causes lung fibrosis (28). The surfactant protein C (L188Q SFTPC), which appears in interstitial pneumonia, was employed in those studies. This mutated protein induced ER stress, since the expression of BiP and XBP1 was increased. The intra-tracheal injection of bleomycin in the L188Q SFTPC-expressing mice resulted in lung fibrosis. Since those mice demonstrated increased apoptotic death of the alveolar epithelial cells, as well as fibrotic lungs, it was assumed that ER stress may cause lung fibrosis (11). Interestingly, the pulmonary UPR is activated due to a plethora of parameters with associated lung fibrosis. Among those factors are infections with viruses, as well as aging (29).

Idiopathic pulmonary fibrosis (IPF) is characterized by elevated levels of GRP78. Cells were exposed to TGF-β1 to evaluate the effects of ER stress in the α-smooth muscle actin and collagen type I expression in the fibroblasts. This growth factor elevated the expression of three different ER stress markers, namely, GRP78, XBP-1, and ATF6α. Moreover, it exerted similar effects in the expression of α-SMA and collagen type I (30).

Chronic obstructive pulmonary disease (COPD) and chronic bronchitis are characterized by increased oxidative stress (31). Inflammatory processes, apoptotic phenomena, and autophagy are major factors in the devastations due to COPD (32). Although PERK induction has been strongly involved in the development of COPD, it appears that the involvement of IRE1 and ATF6 toward those events has not been delineated yet (33).

The lungs of individuals exposed to the smoke of cigarettes, as well as those of COPD patients, express elevated BiP levels. Thus, it was proposed that pulmonary BiP secretion may explain the increase in serum BiP of COPD patients. Researchers measured BiP levels in the bronchoalveolar lavage fluid (BALF) of chronic and nonsmokers. It was revealed that BiP was increased in the case of smokers and that the human airway epithelial cells of the smokers secreted BiP compared to healthy individuals. Thus, it was suggested that BiP expression in the lungs may serve as a marker of lung injury due to smoke (34).

Inhibition of ATF6 activation opposes the development of PAH. ATF6 activation causes upregulation of the neurite outgrowth inhibitor (Nogo), a protein responsible for ER maintenance. Nogo induction causes ER disruption to inhibit key calcium-sensitive enzymes involved in the progression of pulmonary hypertension (PH). Mice lacking Nogo appear to be phenotypically normal but are resistant to hypoxia-induced PAH (35).

Endothelial cell (EC) inflammation and barrier dysfunction are critical events in the pathogenesis of acute lung injury (ALI) and acute respiratory distress syndrome (36). Preconditioning of human pulmonary artery endothelial cells (HPAEC) to ER stress alleviated EC inflammation. BiP silencing inhibited NF-κB activation. Moreover, pre-exposure to SubAB resulted in lessened expression of several inflammatory mediators. Interestingly, BiP suppression resulted in the restoration of endothelial permeability. Those findings suggest the important role of BiP in the NF-κB-mediated endothelial inflammation (37).

A significant risk factor for ARDS development is obesity. It has been shown that in obese mice, there is significant endothelial dysfunction in the lungs. Those obese rodents exert an enhanced susceptibility to LPS-induced lung damage. Moreover, they present increased ER stress in their lung cells, similar to those changes due to exposure to tunicamycin. ER stress reduction protected those obese mice against LPS-induced ALI (38).

Increased ER stress levels due to MAPK induction have been involved in the development of cystic fibrosis (CF). This atypical UPR activation was not associated with the PERK-eIF2α induction. However, the CF cells exerted a hyper-inflammatory phenotype. Salubrinal, a selective elF2a inhibitor, weakened the inflammatory responses due to flagellin (immune activator) and Pseudomonas aeruginosa. Moreover, the abundance of IL-6 was dependent on the activation of the p38 MAPK pathway (39).

ER stress contributes to the development of bronchial asthma by regulating NF-κB activation. The ER stress markers in peripheral blood mononuclear cell and BALF fluid from asthmatic patients were elevated compared to that of healthy individuals. In mice, the chemical chaperone 4-phenylbutyric acid, which alleviates ER stress, counteracted the translocation of NF-κ? Moreover, it reduced the levels of inflammatory cytokines and dendritic cell infiltration (40).

In malignant tissues, it is not certain whether UPR suppresses tumor growth or protects tumor cells by facilitating adaptive survival responses (41, 42). In cases of breast cancer, UPR activation appears to be protective and has been associated with anti-estrogen resistance. On the other hand, hyperactivation of the anticipatory UPR pathway in cancers converts it from cytoprotective to cytotoxic (23). In conclusion, the previously mentioned studies support the deteriorating role of UPR induction in the lungs and support the concept that effective inhibition of ER stress may serve as a promising therapeutic strategy toward lung disease.

## UPR Induction Promotes Lung Health

In the porcine reproductive and respiratory syndrome, IRE1α activities due to ER stress were associated with TNF-α production. Indeed, PERK suppressed this growth factor and protected against heart failure and lung remodeling (43). PERK KO mice were used to test the effects of experimental induced lung fibrosis. TAC (transverse aortic constriction)-induced congestive heart failure (CHF) caused an increase in FM small arteries in all groups. However, the number of those arteries was larger in the PERK KO mice than in the control mice. Furthermore, both wild-type and PERK KO mice exhibited a significant decrease in NM small arteries. However, the number of NM small arteries was lower in PERK KO. Thus, the TAC-induced lung fibrosis was exacerbated in the lungs of PERK KO animals (44).

In knock-in mice that express a mutant BiP, it was revealed that this protein plays a critical role in the surfactant of the lungs. The expression of this mutant BiP in newborns resulted in respiratory failure. Indeed, UPR failure to increase lung susceptibility to hypoxia and ischemia leads to neonatal respiratory failure, indicating that the UPR of alveolar cells is crucial for normal ER function and overload due to regular growth and development (45).

CHOP induction due to double-stranded RNA-activated protein kinase (PKR) suppresses lung injury due to hyperoxia. MLE-12 cells (lung epithelial cells) were exposed to hyperoxic conditions, which in turn elevated both CHOP expression and PKR activation. Interestingly, PKR suppression reduced the hyperoxia-induced CHOP expression. Moreover, hyperoxia induced both lung PKR phosphorylation and CHOP. Mice that did not express CHOP (CHOP KO mice) presented respiratory dysfunction due to lung edema and increased endothelial permeability. Thus, this study supported the protective role of CHOP in the hyperoxia-induced lung dysfunction (1).

Newborns with respiratory stress, when exposed to hyperoxic conditions, are susceptible to the development of lung injury and bronchopulmonary dysplasia. ERp57 is an ER thiol oxidoreductase, which is recruited to substrates through its association with calnexin and calreticulin. Both are molecular chaperones. Calnexin retains unfolded or unassembled N-linked glycoproteins in the ER. Calreticulin participates in various cellular processes, and it was first identified as a Ca^2+^-binding protein. Knockdown of ERp57 was associated with an increase in BiP expression levels and protects against apoptosis due to tunicamycin and hyperoxia (46).

Rhinovirus (RV) infection may result in CF. Studies detected an induction of BiP and CHOP in CF lungs after RV infection. UPR induction after treatment with pharmacologic UPR inducers prior to RV infection protected the cells against cell death. Hence, it was suggested that UPR induction may control respiratory virus replication (47).

DnaJ 4 (ERdj4) is a BiP cochaperone. It removes misfolded proteins from the ER lumen when the cells are exposed to increased levels of toxic factors. A mutated form of ERdj4, which is not functional, caused the death of mice. Those fatalities occurred because of hypoglycemia, associated with abnormal growth. The animals that did not die exerted levels of constitutive ER stress in several tissues, including the lungs. Those studies suggested the important role of BiP in lung survival (48).

GHRH antagonists are UPR inducers and have been recently shown to support endothelial barrier function by suppressing major inflammatory pathways (i.e., ERK1/2, JAK2/STAT3) (49) as well as by deactivating cofilin (50). The GHRH antagonist MIA-602 inhibited fibrosis and inflammation in mice subjected to bleomycin. All the animals inflicted with bleomycin were severely inflamed and presented respiratory abnormalities. Remarkably, the GHRH antagonist MIA-602 counteracted those effects both *in vivo* and *in vitro* and suppressed the abundance of major inflammatory markers. That antagonist has also prominently suppressed multiple inflammatory genes (51).

It was recently shown in lung cells that P53 opposes the LPS-inflicted lung endothelial barrier dysfunction, by mediating the RhoA/Rac signaling (36, 52, 53). UPR modulation in lung cells affected P53 expression levels in a positive manner (54). UPR induction elevated the P53 expression levels, while UPR suppressors reduced them. Thus, it was speculated that P53 possibly protects the lung vasculature against ALI/ARDS, at least in part, by inducing UPR (55). Furthermore, the inhibition of Hsp90 has been shown to induce UPR (56). Those compounds induce P53, which in turn orchestrates robust anti-inflammatory responses (57–59).

Remarkably, UPR exerts a protective role in other tissues than lungs. Norartocarpin (NOR) induced the activity and stability of Nrf2, which has been shown to alleviate pathological outcomes of ER stress. Those effects ware associated with the manipulation of the following molecular pathways, which are crucial for cellular existence and survival: ERK1/2, phosphatidylinositol-4,5-bisphosphate 3-kinase, protein kinase C, and PERK. The latter kinase, as previously mentioned, is an essential component of the UPR (60).

IRE1 has been shown to mediate the protective effects of LPS on myocardial ischemia–reperfusion injury. The LPS was administered in low doses. Rodents (rats) and cells were pretreated with a low dose of LPS prior to myocardial I/R injury. Unexpectedly, the administration of LPS at low doses did not harm the cells and did not significantly affect the animals. Indeed, it attenuated myocardial apoptosis. Proteins closely associated with IRE1 were increased during I/R injury. Such proteins are BiP, phospho-ASK1, and phospho-JNK. However, those effects were reduced by the LPS treatment (14).

The crucial role of CHOP against inflammation was reflected in the fact that CHOP deficiency in mice (CHOP KO) resulted in more significant kidney injury due to LPS, as compared to the wild-type animals. Those effects were associated with increased inflammation responses. Moreover, those LPS-inflicted animals contained a higher amount of renal neutrophil infiltrates compared to the wild-type counteracts. In the kidneys of those mice, increased NF-κB activation and significant upregulation of pro-inflammatory genes were detected. Moreover, LPS treatment elevated the CHOP expression levels in wild-type mice (glomeruli, podocytes). Thus, the authors speculated that the increased CHOP in the kidneys of mice may be protective against AKI and oppose inflammation (61).

It was recently reported that the noncanonical mitochondrial UPR impairs placental oxidative phosphorylation in early-onset preeclampsia. Thus, understanding mitochondrial stress may provide new insights regarding that pathology (62). The beneficial activities of GHRH antagonists in breast and prostate experimental models of disease have been associated with the induction of UPR and P53 (50, 55).

## Conclusions

The role of UPR in the lungs is diverse. Based on the previously referenced literature, it is evident that UPR may protect the lungs against human diseases or potentiate the intensity of various pathologies. In our opinion, the majority of the researchers are focused on the pathophysiological outcomes of UPR activation in lung microvasculature. Thus, most of the studies are targeted toward the suppression of UPR activation or alternatively aim to induce severe ER responses, which inevitably cause cell death.

In a similar fashion, P53 can kill the cells upon intense environmental and extracellular factors (36, 63), but its mild induction delivers protective effects against lung hyperpermeability (58). An increased production of reactive oxygen species may either promote lung pathologies or kill the cancer cells (64). Based on recent observations, we feel that the delineation of UPR signaling in the lungs may reveal new therapeutic possibilities toward lung disease and reveal new targets for lung disease, including ALI/ARDS (56, 65, 66). Experimental murine models of ALI/ARDS with endothelial specific mutations in control of the activation/deactivation of UPR branches shall be used to investigate the unexplored depths of the UPR universe and reveal potential therapies against respiratory dysfunctions.

## Data Availability Statement

The original contributions presented in the study are included in the article/supplementary materials, further inquiries can be directed to the corresponding author/s.

## Author Contributions

The author confirms being the sole contributor of this work and has approved it for publication.

## Conflict of Interest

The author declares that the research was conducted in the absence of any commercial or financial relationships that could be construed as a potential conflict of interest.
